# Effects of fentanyl, midazolam and their combination on immune function and mortality in mice with sepsis

**DOI:** 10.3892/etm.2015.2227

**Published:** 2015-01-28

**Authors:** DONG XIAO, DAQUAN ZHANG, DONGMING XIANG, QI LIU, YAN LIU, LEI LV, XUEZHONG XING

**Affiliations:** 1 Second Department of Critical Care Medicine, People’s Hospital of Xinjiang Uygur Autonomous Region, Ürümqi, Xinjiang 830001, P.R. China; 2 Department of Critical Care Medicine, Cancer Hospital, Chinese Academy of Medical Sciences and Peking Union Medical College, Beijing 100021, P.R. China

**Keywords:** midazolam, fentanyl, immune function, mortality, sepsis

## Abstract

The aim of this study was to investigate the effects of fentanyl and/or midazolam on the immune function and mortality of septic mice. Mice were randomly divided into sham-operated, model, fentanyl-, midazolam- and combination-treated groups. The body weights and locomotor activities of the mice were measured, prior to and following surgery, and the mortality rates following surgery were recorded and compared among these groups. The organ weights and the corresponding coefficients were measured and calculated. Leukocyte numbers in peritoneal and thoracic cavity lavage fluid were counted, and the serum levels of the inflammation-related cytokines interleukin (IL)-1β, IL-10, tumor necrosis factor (TNF)-α, procalcitonin (PCT) and C-reactive protein (CRP) were detected by enzyme-linked immunosorbent assay (ELISA). The results demonstrated that the locomotor activities were reduced in septic mice, and medication led to significant declined body weights in these model animals. Importantly, the mortality rates of the septic mice were reduced by fentanyl and/or midazolam, and the histopathological changes were influenced by the medication. Moreover, in the medication-treated groups, the leukocyte numbers in the peritoneal cavity lavage fluid were lower than those in the model group, while the medication slightly increased the numbers of leukocytes in the thoracic cavity lavage fluid. ELISA indicated that the levels of proinflammatory cytokines were reduced by fentanyl and/or midazolam, which may contribute to the beneficial effects of these medications in septic mice. Analgesic and/or sedative drugs could reduce inflammatory responses in septic mice, and immunosedation may have contributed to the improved mortality rates in these models. These results provide a theoretical basis for further clinical studies concerning the treatment of sepsis with these medications.

## Introduction

Sepsis is one of the most common and serious complications associated with a number of diseases; it is systemic inflammatory response syndrome (SIRS) caused by infection with pathogenic microorganisms. SIRS can subsequently develop into severe sepsis, septic shock and even multiple organ dysfunction syndrome (MODS). Patients suffering from sepsis utilize numerous medical resources, and the mortality rate of the disease is high, and is continuing to rise (1).

The pathogenesis of sepsis is very complex, and several medications have been used to alleviate the symptoms. It has been reported that analgesic and/or sedative medications can relieve pain and anxiety in septic patients, and reduce stress responses (such as increased myocardial oxygen consumption, hypercoagulable state and immune suppression), making these patients feel more comfortable and coordinated (2). Specifically, midazolam, a sedative agent, has been found to inhibit the release of interleukin (IL)-6 mediated by IL-1β in mouse glioma cells, affecting the immune function of the central nervous system (3). Moreover, midazolam has been demonstrated to suppress the inflammatory reaction and stress response in patients with gastric cancer following surgery, contributing to postoperative rehabilitation (4). Fentanyl, an analgesic agent, is able to inhibit the expression of proinflammatory cytokines, including IL-6, IL-10 and tumor necrosis factor (TNF)-α, in lipopolysaccharide (LPS)-induced inflammation (5). However, the effects of fentanyl and/or midazolam on inflammation-related cytokines in sepsis have not yet been fully elucidated.

In the present study, mouse models of sepsis were established to investigate the effects of midazolam and fentanyl, as well as their combination, on immune function and mortality. The aim was to provide a theoretical basis for the clinical application of these analgesic and sedative medications.

## Materials and methods

### Animal grouping and modeling

Ninety specific pathogen-free Kunming mice, half male and half female, weighing 20±2.24 g, were included in the study (SCXK Xin2011-0001; Animal Center of Centers for Disease Control of Xinjiang Uygur Autonomous Region, Ürümqi, China). These mice were randomly divided into the sham-operated (n=10), sepsis model (n=20), fentanyl-treated (n=21), midazolam-treated (n=19) and fentanyl/midazolam combination (n=20) groups. The study and the experimental procedures were approved by the Clinical Ethics Committee and the Animal Care Committee of the People’s Hospital of Xinjiang Uygur Autonomous Region (Ürümqi, China).

The cecal ligation and puncture (CLP) method was used for the establishment of the sepsis model, according to a previously described procedure (6). Briefly, after 8 h of fasting prior to surgery, these mice were weighed and then subjected to anesthesia by the intraperitoneal injection of 10% chloral hydrate (3.5 ml/kg body weight). Following abdominal disinfection, an incision along the abdominal midline was made to open the peritoneal cavity. The cecum was ligated with a silk suture at 0.5–1 cm from the end, and a through-and-through cecal puncture was achieved with a needle. The intestines were then placed back into the peritoneal cavity, and the incision was sutured. For the sham-operated group, the surgery was carried out only with flipping of the cecum.

### Drug administration

Drugs were administrated via intraperitoneal injection, at 10 and 2 h before, as well as 3, 9, 15 and 21 h after the surgery. The dose of fentanyl was 0.0005 mg/kg (Renfu Pharmaceutical Co. Ltd., Yichang, China) and that of midazolam was 1 mg/kg (Enhua Pharmaceutical Co. Ltd., Suzhou, China). For the combination group, 0.0005 mg/kg fentanyl plus 1 mg/kg midazolam were administered. In addition, physiological saline was used in the sham-operated and model groups.

### Locomotor activity detection

Locomotor activity was assessed at 3 h before and 15 h after the surgery in each group. Total activities were detected automatically with an autonomous movement instrument (ZZ-6; Chengdu TME Technology Co., Ltd., Chengdu, China). Each mouse was placed in a box (14.2×11.2×11.4 cm) for 5 min in light.

### General observation

After the mice were sacrificed by cervical dislocation, the peritoneal and thoracic cavities were cut open. Pathogenic changes in the organs, including the heart, thymus, lungs, spleen, liver, kidney, intestine, stomach and brain were observed. The organ weights were measured and the corresponding coefficients were calculated, as previously prescribed (7).

### Leukocyte counting

Three hours after the last drug administration, 4 and 2 ml phosphate-buffered saline (PBS; Tecom Science and Technology Co., Ltd., Nanchang, China) was injected into the peritoneal and thoracic cavities, respectively. After gently rubbing the abdomen, the peritoneal and thoracic cavities were cut open to collect the lavage fluid for leukocyte counting analysis with a fully automated blood cell analyzer (BC-2800Vet; Mindray Medical International Ltd., Shenzhen, China).

### Enzyme-linked immunosorbent assay (ELISA)

Blood samples were collected from carotid arteries prior to sacrifice. These blood samples were centrifuged at 1,000 × g for 10 min, and the harvested serum was subjected to the detection of IL-1, IL-10, TNF-α, procalcitonin (PCT) and C-reactive protein (CRP) with ELISA kits (CRP and PCT kits from Shanghai XiTang Biotechnology Co., Ltd., Shanghai, China, and IL-1, IL-10 and TNF-α kits from Shenzhen Dakewe Biotechnology Co., Ltd. Shenzhen, China), according to the manufacturers’ instructions.

### Statistical analysis

Data are expressed as mean ± standard deviation. SPSS software version 16.0 (SPSS, Inc., Chicago, IL, USA) was used for the statistical analysis. χ^2^ test was performed for the comparison of the leukocyte counting analysis, and the Student’s t-test was used for pairwise comparisons. Variance analysis was applied for multiple comparisons. P<0.05 was considered to indicate a statistically significant result.

## Results

### Effects of fentanyl and/or midazolam on body weights, locomotor activities and mortality rates in septic mice

**Figure 1:**
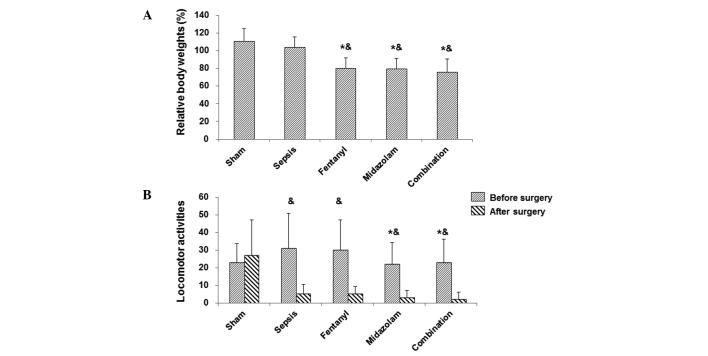
Effects of fentanyl and/or midazolam on body weights and locomotor activities in septic mice. (A) Body weights were measured at 1 h before and 24 h after the surgery in the sham-operated (n=10), model (n=20), fentanyl (n=21), midazolam (n=19) and combination (n=20) groups. The relative changes in the body weight were calculated accordingly. (B) Locomotor activities were recorded at 3 h before and 15 h after the surgery in these groups. ^★^P<0.05 compared with the model group; ^&^P<0.05 compared with before surgery.

Septic mouse models were established by CLP, and the medications were administered at 10 and 2 h before, and 3, 9, 15, and 21 h after the surgery. In the sham-operated group, mice awoke from anesthesia at 2–3 h after surgery, with normal physiological functions, including eating, breathing and bowel movement. However, in the septic models, with or without medications, mice awoke at 3 h after surgery. At 6 h, the model mice begun to suffer from sickness, with decreased motility, lethargy, piloerection, reduced consumption, tachypnea, pyuria, diarrhea and eye exudates. As these symptoms progressed, the mice exhibited shivering, stiff limbs, dyspnea and even neck stiffness, and finally death occurred. In the sham-operated and model groups, body weights were increased following surgery (Fig. 1A). However, in the medication-treated groups (fentanyl, midazolam and their combination), the body weights were significantly declined compared with those prior to the surgery (P<0.0; Fig. 1A). With regard to locomotor activities, there were no significant differences among these groups at 3 h prior to the surgery. At 15 h after the surgery, locomotor activity did not markedly change in the sham-operated group, while in septic mice (with or without medications) the activities were reduced, with no significant differences among these groups (Fig. 1B).

**Figure 2:**
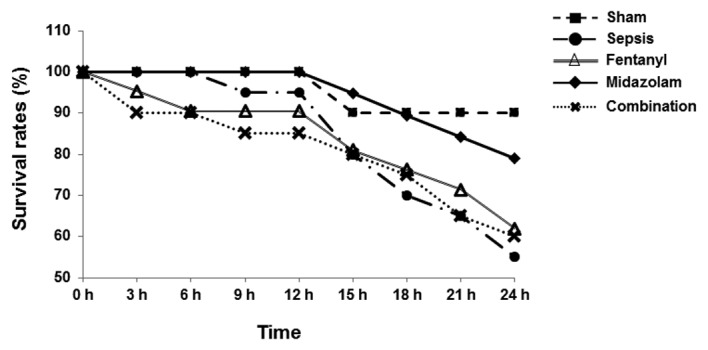
Effects of fentanyl and/or midazolam on the mortality rate of septic mice. The conditions of the mice were monitored and the survival rates were recorded at hourly intervals in the sham-operated (n=10), model (n=20), fentanyl (n=21), midazolam (n=19) and combination (n=20) groups.

To investigate the effects of fentanyl, midazolam and their combination on the mortality of septic mice, the conditions of the mice were monitored and the survival rates were recorded at hourly intervals. As shown in Fig. 2, in the model group, mice started to die at 9 h after surgery. The first death occurred at 3 h after surgery for the fentanyl group, at 3 h for the combination group, and at 15 h for the midazolam group (Fig. 2). Among the mice subjected to CLP, at 24 h after surgery the mortality rate was highest in the model group (45.0%), while the lowest mortality rate was in the midazolam group (21.1%, 4/19; P<0.05). The mortality rates in the fentanyl and combination groups were 38.1 and 40.0%, respectively. These results suggest that the mortality rate of septic mice was reduced by treatment with fentanyl and/or midazolam.

### Impacts of fentanyl and/or midazolam on organ weights and coefficients in septic mice

**Figure 3:**
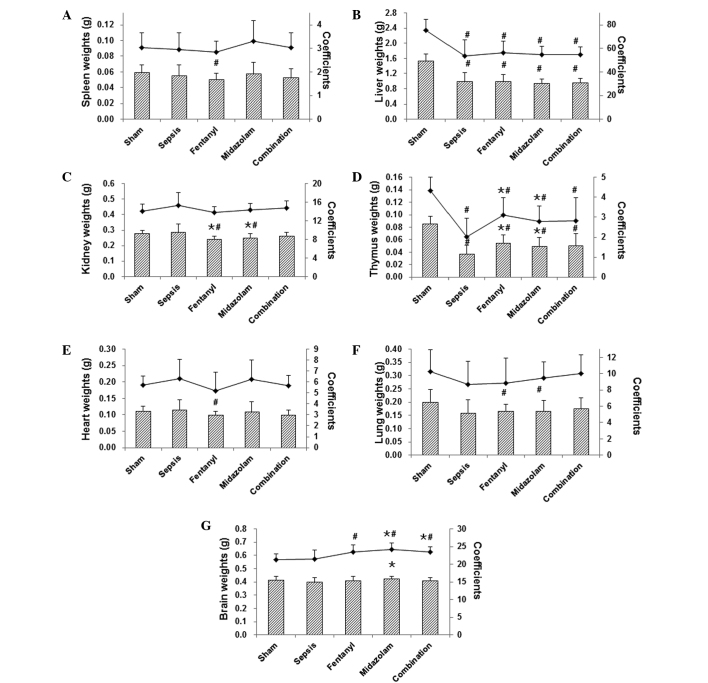
Impacts of fentanyl and/or midazolam on organ weights and coefficients in septic mice. After sacrifice, the weights of each organ were measured (left axis), including (A) spleen, (B) liver, (C) kidney, (D) thymus, (E) heart, (F) lungs and (G) brain in the experimental groups. The corresponding coefficients were also calculated (right axis). ^#^P<0.05 compared with the sham-operated group; ^★^P<0.05 compared with the model group.

To further investigate the effects of fentanyl, midazolam and their combination on the pathology of sepsis, the organ weights were measured and the coefficients were calculated in these mice. Observation of the organs indicated that, in the sham-operated group, no abnormalities were present in the heart, thymus, lungs, spleen, liver, kidney, intestine, stomach or brain. However, in the model group, there were evident bloody exudates in the peritoneal cavity, and distension of the jejunum, swelling of the cecum and gangrene. Livers with uneven or dark color, reddish lungs and meningeal venous congestion were also observed. The weights of the liver and thymus were significantly decreased in septic mice compared with those in the sham-operated group, while the weights of spleen, kidney, heart, lung, and brain did not change markedly. In the medication-treated groups, the administration of fentanyl and midazolam led to reduced kidney and increased thymus weights, while the brain weight was elevated in the midazolam group compared with those in the sepsis model group (Fig. 3).

Concerning organ coefficients, the liver and thymus coefficients were clearly declined in the model group compared with those in the sham-operated group. Following drug administrations, the brain coefficient was significantly elevated for all treatments (Fig. 3G). Furthermore, the thymus coefficient was increased for both fentanyl and midazolam treatments, and the lung coefficient was increased for midazolam treatment and the combination therapy, but no significance was reached (Fig. 3F). These results suggest that the histopathological changes (as indicated by the organ weights and coefficients) were influenced by the administration of fentanyl and/or midazolam in septic mice.

### Effects of fentanyl and/or midazolam on the expression of inflammation-related cytokines in septic mice

**Figure 4:**
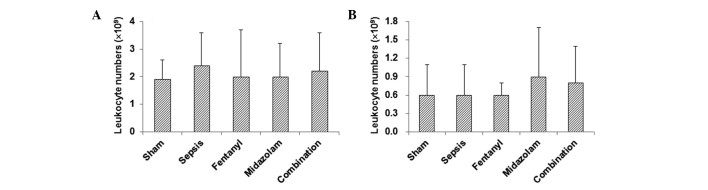
Effects of fentanyl and/or midazolam on the leukocyte numbers in peritoneal and thoracic cavity lavage fluid. At 3 h after the last drug administration, phosphate-buffered saline was injected into the peritoneal and thoracic cavities of the mice After gently rubbing the abdomen, the peritoneal and thoracic cavities were cut open to collect the lavage fluid, and the leukocyte numbers were counted in the (A) peritoneal and (B) thoracic cavity lavage fluid.

To further elucidate the mechanism through which fentanyl and/or midazolam exert beneficial effects in these septic mice, the leukocyte numbers in peritoneal and thoracic cavity lavage fluid were counted, and inflammation was evaluated by detecting the expression of pro-inflammatory cytokines by ELISA. The results indicate that leukocyte numbers in the peritoneal cavity lavage fluid were elevated in the model group compared with those in the sham-operated group, without significant differences (Fig. 4A). In the medication-treated groups, the leukocyte numbers in the peritoneal cavity lavage fluid were lower than those in the model group (Fig. 4A). For the thoracic cavity lavage fluid, there were no significant differences in leukocyte numbers between the sham-operated and model groups, while the administration of fentanyl and/or midazolam slightly increased the number of leukocytes (Fig. 4B).

**Figure 5:**
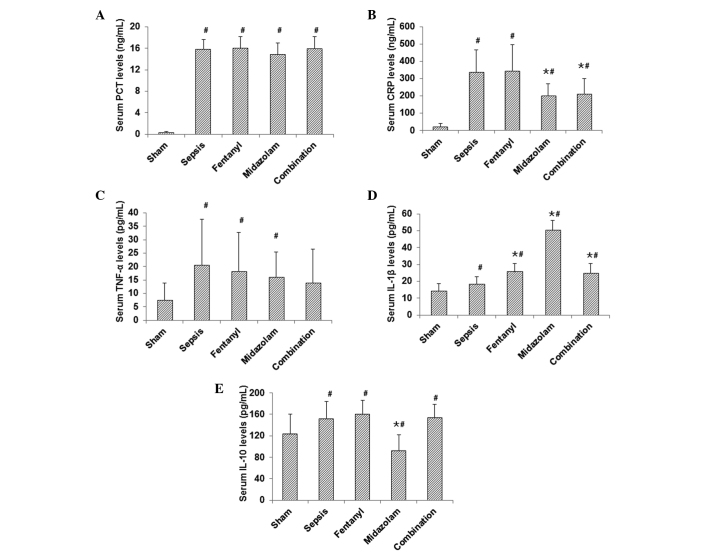
Effects of fentanyl and/or midazolam on the expression of inflammation-related cytokines in septic mice. Serum levels of (A) PCT, (B) CRP, (C) TNF-α, (D) IL-1β and (E) IL-10 were evaluated by ELISA in the sham-operated, model, fentanyl, midazolam and combination groups. ^#^P<0.05 compared with the sham-operated group; ^★^P<0.05 compared with the model group. PCT, procalcitonin; CRP, C-reactive protein; TNF, tumor necrosis factor; Il, interleukin; ELISA, enzyme-linked immunosorbent assay.

Results from ELISA demonstrated that, compared with those in the sham-operated group, the expression levels of PCT, CRP, TNF-α, IL-1β and IL-10 were significantly elevated in the model group (P<0.05; Fig. 5). In the midazolam-treated group, the expression levels of CRP, IL-10, TNF-α and PCT were decreased, while the IL-1β level was increased, compared with those in the model group (P<0.05 for CRP, IL-10 and IL-1β; Fig. 5). In the fentanyl-treated group, the expression level of TNF-α was significantly reduced (P<0.05), while the expression level of IL-1β was significantly increased (P<0.05), compared with those in the model group (Fig. 5). In the combination treatment group, only the expression level of CRP was significantly reduced compared with that in the model group (P<0.05; Fig. 5). These results suggest that the levels of proinflammatory cytokines were reduced by the administration of fentanyl and/or midazolam, which may contribute to the beneficial effects of these medications in septic mice.

## Discussion

Fentanyl and midazolam are the most commonly clinically used analgesic and sedative drugs. The concept of immunosedation in the treatment of sepsis has been proposed (8), based on the finding that midazolam and dexmedetomidine can improve the prognosis of septic mice (9). Accordingly, in the present study, mouse models of sepsis were established, to investigate whether analgesia/sedation with fentanyl and/or midazolam could influence the expression of cytokines and the prognosis of septic mice. It was found that, at 15 h following the modeling surgery, the locomotor activity did not clearly change in the sham-operated group, while in septic mice (with or without medications), the activities were reduced. In particular, the reduction of locomotor activity in the midazolam group was less than that in the fentanyl group, indicating that within 24 h after surgery, it may be necessary to apply the analgesic agent prior to the sedative, and that the optimal outcome may not be achieved with sedative alone.

Sepsis, induced by infection, can cause damage to organs, including the liver, kidney, lung, and brain, leading to immune disorders, forming a vicious cycle resulting in enhanced actions of septic bacteria and related toxins (1). The results of the present study demonstrated that the organ weights and coefficients of the liver and thymus were significantly reduced, while the spleen weight and coefficient were not markedly changed in septic mice compared with those in sham-operated mice. As the thymus is the main organ for T-lymphocyte development, the CLP-induced sepsis models used in the study may be associated with immune dysfunction. The organ weights and coefficients of the thymus were significantly elevated in fentanyl and/or midazolam groups compared with those in the model group, indicating that analgesia/sedation may regulate cellular immunity. Notably, the organ weights and coefficients of the brain and lung were also influenced by the medications, possibly indicating the swelling of the brain and lung induced by hypotension in the model mice. These results suggest that, in the clinical use of midazolam, hypotension should be carefully monitored and appropriately treated to avoid damage to the brain and lung.

Inflammation has been associated with the pathogenesis of sepsis. A previous study has shown that midazolam can inhibit the inflammation and stress response in patients with gastric cancer subsequent to surgery, contributing to their rehabilitation (4). In the present study, the administration of analgesic and sedative drugs decreased the numbers of leukocytes in the peritoneal cavity lavage fluid in septic mice, indicating that these drugs may inhibit the inflammatory response in sepsis. It is widely accepted that infection can trigger an inflammatory cascade in the body, causing systemic immune dysfunction and finally leading to sepsis (10). Numerous factors are involved in the inflammatory process in the development of sepsis. In line with this, the results of the present study demonstrated that, compared with those in the sham-operated group, the expression levels of inflammation-related cytokines, including IL-1β, TNF-α and IL-10, were elevated in the septic mice.

IL-1β and TNF-α are proinflammatory cytokines, initiating the inflammatory response in the body. IL-1β and TNF-α are also able to induce hypotension and subsequent hemodynamic instability (11,12). It has been reported that in patients with meningitis, IL-1β expression levels are negatively correlated with prognosis, that is, a higher IL-1β expression level results in a poorer prognosis (13). However, the association between IL-1β expression level and prognosis in patients with sepsis has not yet been established (14). The present study found that the administration of analgesic and/or sedative drugs markedly reduced the expression level of TNF-α in septic mice, indicating that analgesia/sedation reduce the inflammatory response, which may be associated with the improved mortality rate in the medication-treated groups compared with the model group. These results are consistent with the findings from Babcock *et al* (15). Moreover, Qiao *et al* (9) found that the application of midazolam and dexmedetomidine significantly reduced the mortality rate of septic mice, which may be associated with decreased levels of IL-6 and TNF-α. Wu *et al* (5) also reported that fentanyl caused a marked reduction in the levels of IL-6 and TNF-α in human peripheral blood induced by LPS. These results demonstrate the mortality-reducing effects of fentanyl in septic mice.

IL-10 is one of the important immunoregulatory and anti-inflammatory cytokines, mainly produced by mononuclear macrophages as well as T and B lymphocytes. The major biological effects of IL-10 include inhibiting the antigen-presenting function of macrophages and suppressing the synthesis of TNF-α and other cytokines. IL-10 can reduce excessive inflammation in sepsis, and IL-10-deficiency leads to multiple organ failure and increased mortality rates in mice (16). However, the excessive release of anti-inflammatory and immunosuppressive factors may make it difficult to control the infection. Hypersecretion of the anti-inflammatory mediator IL-10 may cause excessive immunosuppression, and reduce the body’s resistance to infection, inducing secondary infection and even leading to sepsis. In the present study, the expression level of IL-10 was significantly higher in the model group than in the sham-operated group. In medication-treated groups, only treatment with midazolam clearly decreased the IL-10 expression, indicating that midazolam is able to suppress the hypersecretion of IL-10, reduce immune dysfunction and thereby improve the survival rate in septic mice.

C-reactive protein (CRP) is an acute phase protein, which is produced by cells in the liver, kidney and lung under pathological conditions. CRP is a sensitive marker for non-specific inflammation in the body, and therefore is also a highly correlated index of sepsis (17). CRP levels reflect the severity of tissue injury and infection. Continuously high-levels of CRP may stimulate SIRS and high metabolic reactions, resulting in malnutrition and a lack of structural proteins, exacerbating the disease process. In the present study, the serum level of CRP was significantly higher in the model group than in the sham-operated group, indicating severe inflammatory responses in these models. The administration of midazolam alone or in combination with fentanyl markedly downregulated the expression of CRP, indicating inhibition of the inflammatory response in sepsis. PCT is the 116-amino precursor to calcitonin, which has been associated with the pathogenesis of sepsis and other inflammatory injuries. It has been reported that the PCT level is positively correlated with the severity of sepsis (18). In the present study, the PCT level was lower in the midazolam group compared with that in all other septic groups, with or without medications, which may be associated with the lower mortality in this group.

In conclusion, the results demonstrated that analgesic and/or sedative drugs are able to reduce inflammatory responses in septic mice, and that immunosedation contributes to the improved mortality rate in these models. This study investigated the modulating effects of analgesia/sedation on the immune function in sepsis, providing a theoretical basis for further clinical studies concerning the treatment of sepsis. Further studies are required to elucidate the detailed mechanism(s) through which analgesia/sedation exert immune-regulating effects.
